# Transient Receptor Potential Vanilloid in the Brain Gliovascular Unit: Prospective Targets in Therapy

**DOI:** 10.3390/pharmaceutics13030334

**Published:** 2021-03-04

**Authors:** Huilong Luo, Xavier Declèves, Salvatore Cisternino

**Affiliations:** 1 INSERM, U1144, F-75006 Paris, France; hluo67@wisc.edu (H.L.); xavier.decleves@parisdescartes.fr (X.D.); 2 Faculté de Pharmacie, Université de Paris, INSERM, UMRS-1144, Optimisation Thérapeutique en Neuropsychopharmacologie, F-75006 Paris, France; 3 Department of Chemical and Biological Engineering, University of Wisconsin, Madison, WI 53706, USA; 4 Biologie du Médicament et Toxicologie, Assistance Publique Hôpitaux de Paris, AP-HP, Hôpital Universitaire Cochin, F-75014 Paris, France; 5 Service Pharmacie, Assistance Publique Hôpitaux de Paris, AP-HP, Hôpital Universitaire Necker—Enfants Malades, F-75015 Paris, France

**Keywords:** blood-brain barrier, cannabidiol, membrane proteins, neurovascular unit, transient receptor potential cation channels

## Abstract

The gliovascular unit (GVU) is composed of the brain microvascular endothelial cells forming blood–brain barrier and the neighboring surrounding “mural” cells (e.g., pericytes) and astrocytes. Modulation of the GVU/BBB features could be observed in a variety of vascular, immunologic, neuro-psychiatric diseases, and cancers, which can disrupt the brain homeostasis. Ca^2+^ dynamics have been regarded as a major factor in determining BBB/GVU properties, and previous studies have demonstrated the role of transient receptor potential vanilloid (TRPV) channels in modulating Ca^2+^ and BBB/GVU properties. The physiological role of thermosensitive TRPV channels in the BBB/GVU, as well as their possible therapeutic potential as targets in treating brain diseases via preserving the BBB are reviewed. TRPV2 and TRPV4 are the most abundant isoforms in the human BBB, and TRPV2 was evidenced to play a main role in regulating human BBB integrity. Interspecies differences in TRPV2 and TRPV4 BBB expression complicate further preclinical validation. More studies are still needed to better establish the physiopathological TRPV roles such as in astrocytes, vascular smooth muscle cells, and pericytes. The effect of the chronic TRPV modulation should also deserve further studies to evaluate their benefit and innocuity in vivo.

## 1. Introduction

The brain gliovascular unit (GVU) consists firstly of brain microvascular endothelial cells (BMECs) forming the blood–brain barrier (BBB) and secondly of the neighboring surrounded “mural” cells (e.g., pericytes) and astrocytes [1]. The BBB is a tight and non-fenestrated endothelial monolayer which helps to maintain brain homeostasis by controlling cellular and molecular permeability from the blood to the brain parenchyma and vice versa [2]. The highly sealed tight junctions expressed between BMECs exert a “physical” barrier which greatly limits paracellular diffusion [3]. The transcellular diffusion of compounds across the BBB is further controlled by reduced vesicles trafficking and by the expression of selected efflux transporters from the ATP-binding cassette (ABC) superfamily which transport them back into the capillary lumen [4,5]. Some nutrients and/or xenobiotics transporters from the solute carrier (SLC) superfamily are also specifically expressed in the BBB, allowing transcellular transport of substrates to the brain such as glucose, amino acids, or drugs [5,6]. The role of the BBB in the control of leukocyte trafficking under physiological and pathological conditions is also a specific feature of the BMECs [7].

Currently, a growing body of evidence has shown that BBB, as a modulatory interface, is also regulated by signaling processes involving the surrounding cells of the brain parenchyma known as astrocytes and perivascular cells referred to as mural cells (e.g., pericytes), which all together form the GVU [8]. Astrocytes, the most abundant glial cells of the brain, are multipolar cells whose morphology allows the integration of both the neuronal and vascular signals supporting the neurovascular coupling that helps to adapt the local blood flow supply to the neuronal activity demand. Astrocytes are recognized as an important GVU partner not only in modulating the functionality of the brain endothelial cells, but also in regulating the blood flow [9]. Besides the astrocytes, the interplay with the pericytes embedded in the basal lamina plays an ill-defined role in the organization of the perivascular complex, but several recent studies highlight also the role of the pericyte in the modulation of the functions of the BBB/GVU [10,11,12]. Under pathological conditions, signaling pathways between the BBB and the neighboring cells of the GVU have been evidenced to serve as an emerging target in modulating and restoring BBB dysfunctions to preserve brain homeostasis [8,13].

GVU/BBB dysfunctions, as a cause or consequence, can be found in various central nervous system (CNS) diseases. Indeed, several chemical/physical stimuli from the blood and/or brain parenchyma compartments can modulate the BBB features and lead to changes in the vascular flow, immune cell trafficking, and/or the tight junction integrity. Such events may further participate in the development of neurological disorders such as multiple sclerosis [14]. The modulation of the BBB features, in particular altering the permeability to a particular molecule or to a cell, is observed in a variety of neuropsychiatric, vascular, metabolic, and immunologic diseases, and also in cancers. Various CNS diseases can also lead to a regional BBB disruption, which exposes the sensitive neural environment, alters the brain homeostasis, and in turn worsens the symptoms [15]. For example, a series of pathophysiological signaling molecules related to inflammation and oxidative stress released during cerebral ischemia–reperfusion can increase BBB permeability [16], which in turn causes potentially fatal symptoms such as cerebral edema and hemorrhage [17]. Clinical observations showed that disruption of BBB integrity is associated with a poor outcome and prognosis of ischemic stroke [18]. Alongside stroke, BBB disruption and dysfunction happen in various CNS diseases, including, but not limited to, traumatic brain injury (TBI), glioma/cancers, epilepsy, neurodegenerative diseases such as Parkinson’s diseases, and Alzheimer’s disease [19]. In all cases, any strategy that preserves or rescues BBB integrity/dysfunction is of importance in the treatment and prognosis of BBB-related CNS diseases. Consequently, more attention has been paid to focus on the BBB/GVU as a therapeutic target in treating CNS diseases [3,20].

Calcium dynamics in BMECs have been proven to play a major role in determining BBB properties [21]. In the past decades, transient receptor potential (TRP) channels have been identified as important mechanisms in regulating at least the transmembrane flux of inorganic ions such as Ca^2+^, as well as other inorganic cations such as Mg^2+^, Na^+^, and K^+^ [22], making them participate in various pathophysiological processes including the modulation of BBB properties. Human TRP channels consist of 27 isoforms divided into six subfamilies according to their sequence homology: ankyrin (TRPA), canonical (TRPC), melastatin (TRPM), mucolipin (TRPML), polycystin (TRPP), and vanilloid (TRPV).

The primary structure of TRPV1–4 consists of six transmembrane segments with a pore region between the fifth and sixth segment [23]. The pore will be opened and allow the Ca^2+^ flowing into the cytoplasm upon activation [23]. TRPV respond to a wide range of stimulations, such as pH, heat, membrane voltage alternation, surrounding irritants, hypo/hyperosmotic stress, and mechanical stretch [24]. TRPV channels have been one of the most aggressively pursed drug targets, with several drug candidates targeting directly TRPV and entering clinical trials or even already approved as drugs in treating pain, epilepsy, or heart failure [25,26,27,28,29,30,31,32,33,34,35,36,37,38] (Table 1). Indeed, the main and first knowledge of TRPV functions and their ligands come from their somatosensation properties (e.g., temperature sensing, nociception/pain, chemosensing) and expression in sensory neurons [39]. The pharmacology of TRPV was first illustrated with natural compounds already used in traditional medicine such as menthol, eucalyptol, and capsaicin (CAP). For example, CAP, a selective TRPV1 agonist derived from chili peppers, has been approved by U.S. Food and Drug Administration (FDA) in treating neuropathic pain [40]. Recently, more studies have evidenced TRPV expression and their ability in modulating the functionalities of non-sensory cells using various chemical agonists and antagonists [41,42]. The role of TRPV channels in peripheral endothelial cells where they possibly contribute to vascular physiology are also reported [43], although TRPV functions and signaling in endothelial cells could differ from what it is observed in other cells. Interestingly, the functions of TRPV1 [44], and more recently for TRPV2 [45] and TRPV4 [46], in brain endothelial cells forming the BBB showed their participation in modulating BBB properties. Interestingly, their localization at the vascular interface enhances their role in mechanotransduction mechanisms and possibly responses to vascular/shear stress (e.g., migration, adhesion), as TRPV2 and TRPV4 are also known to be associated with actin/cytoskeleton proteins [47]. In this review, we will summarize the role of TRPV channels in the GVU with a particular focus on the BBB and discuss their physiological and/or potential as drug targets in CNS diseases. 

## 2. Overview of TRPV1–4 Expression in Brain Regions 

TRPV channels have six isoforms further divided into two subgroups: TRPV1–4 and TRPV5–6. TRPV1–4 are also called “thermo-sensitive” TRPVs due to their ability to be activated by different heat temperatures [48]. In contrast, TRPV5 and TRPV6 demonstrate significantly different properties compared with TRPV1–4 and are mostly expressed in renal and intestinal epithelia [49], with no evidence of their expression in the CNS [50]. TRPV1–4 expression is quite heterogeneous and region-specific in the brain [50,51,52] (Table 2). For TRPV1, it has been previously reported to be widely expressed throughout the brain [50]. However, based on a highly sensitive method applying gene editing techniques to modify the genetic locus of Trpv1 in two types of reporter mice, a study found a high region-selective expression of Trpv1 throughout the brain, such as the caudal hypothalamus, the dorsal motor nucleus of the vagus, mesencephalic trigeminal nucleus, and parabrachial nucleus [51]. Absent or little expression of Trpv1 was found in the other brain regions [51]. For Trpv2, it has been showed to be intensively expressed mainly in several rat brain regions, such as the hypothalamus, the nucleus of the solitary tract, hypoglossal nucleus, and the rostral division of the ventrolateral [52]. Although TRPV3 mRNA transcripts were detected in the brain at low levels, protein expression was not detected throughout the mouse brain [53]. Normally, TRPV3 has the most abundant expression in cells surrounding hair follicles and in skin keratinocytes, where it plays a vital role in the skin physiology and cutaneous sensation. Gain-of-function mutations in human TRPV3 can lead to the Olmsted syndrome, which is characterized by a severe periorificial and palmoplantar keratoderma without affecting cerebral functions [54,55], suggesting a lack of function for TRPV3 in the brain [53]. Similar to TRPV2, TRPV4 is also widely expressed throughout the brain including hippocampus, hypothalamus, cerebellum, lamina terminalis, and optic chiasm, as well as a special enhanced expression in the olfactory placodes [50,56,57]. However, until now, it is still largely unknown which types of cells were involved and responsible for their expression in a specific brain region [50]. However, some functions and/or expressions were studied in GVU cells [58].

## 3. TRPV1–4 Expression in the GVU

TRPV1–4 are identified in a variety of cell types in mammals including neurons both in the peripheral nervous system or the CNS and in many non-neuronal cell types of the body such as immune cells (e.g., lymphocytes, macrophages, dendritic cells), keratinocytes, and some cells of the GVU such as brain endothelial cells [48]. These observations suggested that these TRPV channels could be both mediators of noxious thermal and chemical stimuli that could produce nociceptive responses and other functions unrelated to neuron firing. In this review, we focus our attention on thermosensitive TRPV1–4 in the GVU and in particular in BMECs (Table 3).

### 3.1. TRPV1–4 Expression at the BBB

#### 3.1.1. TRPV1

Golech et al. have identified the expression of TRPV1 in primary cultured human brain endothelial cells isolated from brain capillaries by RT-PCR and Immunohistochemistry (IHC) experiments using polyclonal antibodies [44]. In rodents, the protein expression of TRPV1 in murine BBB endothelial cell line bEnd.3 was identified by Western blot [65]. Expression of Trpv1 in murine brain capillaries was identified by IHC via co-staining experiments with a CD31 antibody used as a BBB marker [65]. CAP is a potent agonist for TRPV1 [59,60] and exhibits high selectivity for TRPV1 as it does not activate other homologue channels within the TRPV family [68,69]. CAP experiments illustrated the BBB function of Trpv1 which dose-dependently stimulated the Ca^2+^ influx in rat BMECs, whereas capsazepine (CPZ), a selective TRPV1 antagonist, dose-dependently inhibited this Trpv1-mediated Ca^2+^ flux [62].

#### 3.1.2. TRPV2

Trpv2 mRNA were detected in both immortalized brain endothelial cell line bEnd.3 and freshly isolated mouse BMECs [63]. TRPV2 mRNA transcript was also evidenced in human BMECs [46]. Abundant TRPV2 expression was detected by qRT-PCR in the human BMEC cell line hCMEC/D3 and in human primary cultured BMECs isolated from brain biopsies in patients, and furthermore confirmed at the protein level by Western blotting, immunofluorescence, and non-targeted proteomics [45]. TRPV2 function was illustrated in hCMEC/D3 cells, showing that heat or the selective TRPV2 agonist cannabidiol (CBD)-mediated Ca^2+^ influx was reduced both by chemical inhibition with ruthenium red (RR), a non-specific TRP antagonist, or tranilast (TNL), a selective TRPV2 antagonist, and by silencing TRPV2 expression with siRNA experiments [45].

#### 3.1.3. TRPV3

Trpv3 was absent in the mouse brain microvessel endothelial cell line bEnd.3 and in the freshly isolated cerebral capillaries and primary cultured rat BMECs [63], which is consistent with the whole transcriptomic analysis showing the absence of Trpv3 in murine primary BMECs [64], and with a study on brain vascular single-cell transcriptomics [58]. These results are also supported regarding the absence of Trpv3 in rat microvessels and BMECs [66] or human BMECs [44,46] when detected by RT-PCR or q-PCR.

#### 3.1.4. TRPV4

The protein expression of TRPV4 was identified in human BMECs [46]. A TRPV4 agonist, 4α-phorbol 12,13-didecanoate (4αPDD) dose-dependently stimulated the Ca^2+^ influx in human BMECs, which was significantly reduced in human BMECs silenced for TRPV4, further validating TRPV4 functional expression in human BMECs [46]. TRPV4 function was also determined using patch-clamp electrophysiology techniques in freshly BMECs isolated from the brain capillaries of a C57BL/6J mouse [61]. The selective TRPV4 agonist GSK1016790A-stimulated currents can be completely abolished in Trpv4-knockout mice or when co-applying the specific TRPV4 antagonist HC-067047 in BMECs [61].

### 3.2. TRPV1–4 Expression in Astrocytes and Pericytes 

Trpv1 was firstly identified to be expressed in mouse spinal glial cells characterized as astrocytes by IHC at the ultrastructural level [70]. Interestingly, using commercially available TRPV1 specific antibodies, Trpv1 immunohistochemical staining can be seen mainly in the astrocytic perivascular processes, also called astrocyte endfeet in the rat brain [71]. Later studies have further identified that the expression of TRPV1 in astrocytes is functional and involved in various biological processes. For example, TRPV1 was shown to be involved in beta-amyloid-mediated inflammatory responses in primary cultured astrocytes isolated from rats [72], evodiamine-induced autophagy in the human U87-MG astrocyte cell line [73], and astrocyte-derived endogenous production of ciliary neurotrophic factor (CNTF) [74] and IL-1beta migration [72,75]. Stress or injury-induced cytoskeletal reorganization and migration in primary cultured astrocytes isolated from mice were also evidenced [76]. 

The expression of TRPV2 in astrocytes was recently identified by biochemical and functional methods showing that TRPV2 was mainly detected in the plasma membrane of primary cultured astrocytes from mice, which can be activated by a high temperature (>50 °C) and lysophosphatidylcholine, a known endogenous lipid ligand for TRPV2 [77]. Blocking TRPV2 can promote cell proliferation, and synthesis and secretion of a neuroprotective factor named nerve growth factor (NGF) in primary cultured astrocytes isolated from rats [78]. Interestingly, CBD was also evidenced to promote glial differentiation in glioma stem-like cells (GSCs) via a TRPV2-dependent manner [79]. In contrast, Trpv3 was reported to be absent according to RNA sequencing data in isolated primary astrocytes in mice [64] and in a single-cell transcriptomic mice study [58].

The expression of TRPV4 in astrocytes was first identified by Western blot experiments, and confocal microscopy in rats revealed a localization at the plasma membrane [80]. Interestingly, as shown for TRPV1, TRPV4 was also enriched in astrocyte endfeet facing brain vessels, strengthening its importance in vascular/BBB interplay [80]. Importantly, TRPV4-induced Ca^2+^ signaling within the astrocyte endfeet contributes to neuronal activation and mediates neurovascular coupling, further controlling vascular tone and local perfusion in the brain [81,82]. Aquaporin-4 (Aqp4), a water channel enriched in the perivascular astroglial endfeet membrane, is well known to be involved in brain edema formation. Aqp4 causes swelling which triggers Ca^2+^ signaling in astrocytes [83]. An Aqp4/Trpv4 complex was found to be essential in regulating cell volume in mice astrocytes, which emphasized the essential role of Trpv4 in brain edema pathophysiology and astrocyte reactivity following ischemic insult [84,85,86]. Astrocytic TRPV4 was also functional and believed to be involved in the pathogenesis of a number of CNS diseases [84,85,86,87,88]. The expression and activity of Trpv4 in the adult rat hippocampus astrocytes was increased after cerebral hypoxia/ischemia in stroke [86,88]. 

Compared with astrocytes, much less information was reported regarding the expression and function of TRPVs in pericytes. Strong Trpv2 expression in mice brain pericytes was evidenced via single cell transcriptomic studies [58]. Trpv1 immunostaining in pericytes was observed when incubating paraformaldehyde perfused rat brain slices with commercial anti-TRPV1 polyclonal antibody [71]. Trpv4 KO mice demonstrate less pericytes coverage surrounding brain capillaries, suggesting a possible role of Trpv4 in mediating the interactions between pericytes and brain endothelial cells [89,90]. In addition, TRPV1, 2, and 4 expression in microglia was confirmed using PCR and immunostaining [91,92,93], which might be also involved in microglia inflammatory responses [93,94,95]. 

## 4. Species Differences in TRPV Expression at the BBB

Hatano et al. have demonstrated the absence of TRPV1–3–5–6 mRNA in commercially cultured human BMECs, in contrast to those of TRPV4 which were 2-fold higher than those of TRPV2 [46,67]. However, TRPV2 was identified as the highest expressed isoform in both primary cultured human BMECs isolated from biopsy and in the human BBB cell line hCMEC/D3 when detected by q-PCR showing the rank order as follows: TRPV2 > TRPV4 > TRPV1, while no TRPV3 mRNA levels were detected [45]. In fact, the mRNA levels of TRPV2 were even much higher than those of the ABCB1 gene encoding the P-glycoprotein, an abundant well-known marker of the human and rodent BBB [4]. Furthermore, a non-targeted proteomic technique to detect TRPV1–4 in human BMECs showed that TRPV2 was the only TRPV isoform detected [45].

The gene expression of Trpv1–4 in the murine BBB cell line bEnd.3 and freshly isolated capillaries was also studied by RT-PCR, showing the existence of Trpv2 and Trpv4 [63], which was close to the expression profile of TRPVs observed in hCMEC/D3 cells and human primary brain microvascular endothelial cells (hPBMEC) [45]. Trpv1–4 expression profile in the rat cortex, rat brain microvessel enriched fractions, and isolated primary cultured rat BMECs (rPBMEC) was also determined by qRT-PCR exhibiting a close Trpv1–4 expression profile in rPBMEC and brain microvessels as follows: Trpv4 > Trpv2> Trpv3> Trpv1 [66]. In addition, a whole transcriptomic analysis revealed a relative equal expression of Trpv2 and Trpv4 (signal value: 0.3 and 0.4, respectively) in murine primary cultured BMECs, while no Trpv1 and Trpv3 mRNA were detected [64]. A single-cell transcriptomic mice study suggests less abundance of Trpv2 in the mouse BBB [58]. Overall, regardless of the possible interspecies differences, TRPV2 and TRPV4 are the two main isoforms in BMECs, with less expression of TRPV1 and absence of TRPV3.

## 5. Functions of TRPV1–4 in Modulating BBB under Healthy and Diseased State

Various molecules possibly disrupting the BBB are potentially formed/released during the pathogenesis of some CNS-affecting diseases [15]. In turn, BBB disruption could exacerbate the permeability to potential harmful vascular compounds which further promote brain damages such as neuron apoptosis and subsequent CNS dysfunctions [96]. For example, ischemia–reperfusion in stroke promotes BMECs injury and leads to BBB disruption, while BBB disruption accelerates the formation of cerebral edema and aggravates the devastating nature of intracerebral hemorrhage (ICH), leading to serious disability in humans and high mortality caused by stroke [97]. The protective effect of TRPV channels in CNS-affecting diseases via preserving or rescuing the BBB integrity has been highlighted recently by in vitro and in vivo studies which were reviewed (see Table 4).

### 5.1. TRPV1

It is known that certain proteins expressed at the BBB, such as those comprising tight junctions (TJ) (e.g., Claudin 5) and transporters (e.g., GLUT1/SLC2A1), might be modulated and maintained by various signals produced by neighboring cells such as astrocytes and pericytes [105]. The lack or dysfunction of the cross-communication between BMECs and neighboring cells might lead to a change of BBB features including permeability alteration [1]. Interestingly, TRPV1 expression was significantly increased by 4.8-fold in human primary BMECs when coculturing with primary cultured astrocytes isolated from the same patient biopsy, suggesting that the interplay between astrocytes and BMECs may modulate BBB TRPV1 expression [45]. 

Hu et al. have demonstrated that Trpv1 activation by CAP resulted in the increased permeability of the BBB by ex vivo experiments in rats, which is blocked by co-treatment with CPZ [62], suggesting the involvement of TRPV1 in modulating BBB permeability. Interestingly, the gene expression of Trpv1 was increased post stroke, and Trpv1 inhibition exhibits some neuro-protective effects during brain ischemia in mice [106]. Mechanical stress performed in the murine brain endothelial cell line bEnd.3 which mimics BBB injury during TBI was shown to increase Trpv1 protein expression [65]. Similar results have been observed in the peri-contusional area of murine brain post TBI, in which the increased Trpv1 protein expression could be reversed by pretreatment with the Trpv1-specific antagonist CPZ [65]. Some endocannabinoids that normally target cannabinoid receptor type 1 (CB1) and/or type 2 (CB2) [107], such as 2-arachidonoyl-glycerol (2-AG) and anandamide (ANA), were reported as endogenous agonists of TRPV1 [44,108]. Golech et al. have found that endocannabinoids, including 2-AG, anandamide (ANA), and methanandamide (m-ANA), stimulated Ca^2+^ influx in human BMECs, which was dose-dependently blocked by the specific TRPV1 antagonist CPZ [44,102]. This is consistent with previous data showing that ANA and its derivatives also activate TRPV1 in hTRPV1-transfected human embryonic kidney (HEK293) cells [109]. Furthermore, Ca^2+^ influx mediated by 2-AG induces phosphorylation of the vasodilator-stimulated phosphoprotein (VSAP) and alternation of cytoskeleton (i.e., actin and vimentin) via activating TRPV1, which is involved in the brain microvessels vasodilation and potentially endothelial-dependent modulation of BBB permeability [44,102]. It is also reported that CPZ totally blocked the decreased BBB permeability stimulated by endocannabinoids ANA and oleoylethanolamide [110]. All these data suggested the involvement of TRPV1 in modulating BBB permeability, making it a possible therapeutic target in various CNS diseases involving this BBB dysfunction.

The endogenous TRPV1 agonist ANA administered into the right lateral cerebral ventricle was evidenced to mediate the formation of cerebral edema, which was greatly reduced by co-administration of CPZ [98]. Hu et al. also have showed that CPZ (1 µmol/kg, IP) reduced the exacerbated increase in brain capillaries permeability in an ischemia–perfusion rat model obtained after injection of degradable starch microspheres [62]. The protective effect of CPZ on the BBB was also demonstrated by Cauden et al., demonstrating that CPZ decreased the post-ischemic increase in BBB permeability to around one third [99]. Yang et al. also found that inhibition of TRPV1 with CPZ decreased vasogenic brain edema and BBB disruption as measured with Evans blue cerebral extravasation and preserved TJ) upon brain injury, reinforcing the interest of using TRPV1 antagonists as a potential therapeutic approach to protect the BBB against such disruption [65].

**Figure 1:**
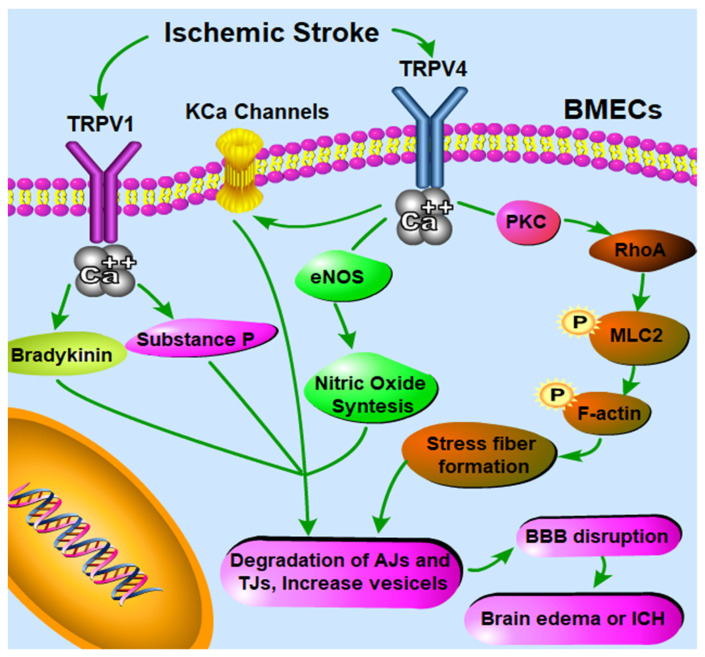
Schematic illustration describing the possible involvement of Transient Receptor Potential Vanilloid (TRPV) in the context of blood brain barrier (BBB) function under ischemic stroke according to current knowledge. TRPV1 antagonism can block bradykinin and Substance P release mediated BBB disruption during cerebral ischemia [62]. The mechanisms underlying TRPV4-mediated BBB permeability modulation are complex. Firstly, Ca^2+^ influx, which is elicited by activation of endothelial TRPV4 channels, can further activate intermediate and small conductance Ca^2+^-sensitive K^+^ (KCa) channels [114], while KCa channels have been well known to be able to induce BBB opening [115]. Secondly, increased intracellular Ca^2+^ via TRPV4 activation can also significantly induce the endothelial nitric oxide synthase (eNOS) phosphorylation [105], and activated eNOS produces more nitric oxide (NO), further activating soluble guanylyl cyclase to produce more cyclic guanosine monophosphate (cGMP); high concentrations of cGMP lead to an increase in the number of pinocytic vesicles containing caveolin-1 and caveolin-2 and BBB permeability [116]. Thirdly, another possible mechanism involved in TRPV4 activation mediated BBB disruption is the formation of stress fibers through the PKCα/RhoA/MLC2 pathway activation. PKCα activation has been involved in TRPV4-regulated endothelial cell permeability alteration [117]. After activation, PKCα can directly interact with RhoA, leading to the rearrangement of the cytoskeleton [118,119]. RhoA further binds to Rho-kinase, which can strongly induce the phosphorylation of myosin light chain 2 (MLC2) and the formation of actin stress fibers [120], thus leading to BBB disruption [121]. Therefore, TRPV4 antagonism is able to inhibit actin stress fibers formation in ischemia, thus preserving/rescuing the integrity of the BBB [101].

The underlying mechanisms in charge of TRPV1-mediated modulation of BBB permeability are, however, complex, and evidence for direct and/or indirect mechanisms is not fully elucidated. Bradykinin is rapidly released during ischemia–reperfusion [111] and further increases BBB permeability via targeting bradykinin receptor 2 [112]. Bradykinin was also found to stimulate TRPV1 when pH decreases, as it occurs in ischemia [113]. TRPV1 antagonism can block this bradykinin-induced increase in BBB permeability after cerebral ischemia underlining multiple and possibly synergetic pathways [62] (Figure 1). It was also suggested that the substance P neurotransmitter could be released upon TRPV1 activation in brain pial vessels and participate in the CAP-induced BBB permeability increase [62] (Figure 1). The phosphorylation of the c-Jun N-terminal kinase (JNK) and p38 mitogen-activated protein kinases (MAPKs) can be down-regulated dramatically in bEnd.3 cells treated with CPZ, resulting in the protective effect on the level of ZO-1 after a stretch injury [65]. This suggests that inhibiting JNK and p38 MAPKs may be of crucial role in mediating the anti-apoptotic effect of CPZ in BMECs to better protect the BBB from disruption [65]. 

To date, no hereditary diseases have been associated with TRPV1 mutation, limiting the gain of knowledge about its function in humans. In Trpv1 knockout (KO) animals, no evidence was given showing whether or not BBB/GVU functionality was maintained. Evidence has suggested an undisrupted brain homeostasis since, in a physiological state, Trpv1 KO mice appeared normal in behavioral tests but have a reduced inflammatory response and higher pain threshold [122]. Trpv1 KO mice experiments demonstrated that Trpv1 activation in astrocytes might be related to the severity of hypoxic ischemic encephalopathy, which is a serious complication at birth [75].

### 5.2. TRPV2

Using an in vitro mouse BBB model, Brown et al. have reported that BBB permeability was increased by activation of the Trpv2 and/or Trpv4 through mediating Ca^2+^ influx [63]. CBD, a potent TRPV2 agonist [123], protected the BBB disruption induced by oxygen-glucose deprivation (OGD) in a human in vitro BBB model consisting of human BMECs co-cultured with human astrocytes via PPARγ and 5-HT1A receptors [100]. Thus, it remained to be determined in this hypoxic model whether TRPV2 was also involved in this BBB protective effect of CBD [100]. A more recent study demonstrated that CBD could modulate the human BBB permeability determined by transendothelial electrical resistance (TEER) via activating TRPV2 [45]. CBD dose-dependently induced cell proliferation and cell migration of hCMEC/D3 cells with an EC50 0.3 ± 0.1 µM, which was fully blocked by TNL or siRNA targeting TRPV2 [45]. Tubulogenesis of hCMEC/D3 cells in 3D matrigel cultures was dramatically increased after CBD treatment, and reversed by TNL. All these results suggest potential endothelial-dependent BBB permeability and protecting effects of CBD on the human BBB through activating TRPV2 [45].

Few studies focused on the involvement of TRPV2 in preserving BBB integrity in ischemic stroke. CBD applied at therapeutic concentrations increased the BBB integrity as measured by TEER value in hPBMEC monolayers cultured in transwells, and this was blocked by TNL indicating the involvement of TRPV2 in this CBD effect [45]. By determining TEER value to detect the BBB leakiness, CBD exhibited a protective role by preventing BBB disruption upon OGD in hPBMEC cells, which is used in vitro as a model of a hypoxia/ischemic stroke [100]. Indirect evidence from Ceprian et al. demonstrated the beneficial effects of CBD in decreasing brain edema and improving cerebral functional recovery in a neonatal rat model bearing ischemic stroke, but the underlying mechanism was not illustrated. It remains unknown whether or not the effect of CBD in preserving BBB integrity was mediated through direct TRPV2 activation in BMECs [124]. The functional expression of TRPV2 was also found in BBB-reacted neighboring cells, such as astrocytes [77], neurons [123], and microglia [125].

Until now, some hereditary diseases were reported to be related to TRPV2 mutation in humans. Trpv2 KO mice were shown to be susceptible to perinatal lethality, but the surviving KO mice exhibited normal behavioral responses to noxious heat and punctate mechanical stimuli [126]. Interestingly, cardiac-specific TRPV2 deficiency in mice showed that TRPV2 was of high importance in maintaining the structure and function of the cardiovascular system as well as in immune cell functions [127]. However, evidence was still lacking in identifying the possible role of Trpv2 in maintaining the cerebrovascular system using KO animals. The possible genetic compensation in gene KO animals is also a limitation of such approach.

### 5.3. TRPV4

TRPV4 responds to alterations in cell volume and acts as an osmotic sensor [128]. In mouse BMECs, hypo-osmotic stimulation can increase the permeability of the BBB via activating of Trpv4 [63]. The specific TRPV4 agonist, GSK1016790A, also induced degradation of adherens and TJ proteins in naive rats, which was reversed by co-treatment with HC-067047 or Trpv4 siRNA, further indicating the participation of Trpv4 in BBB modulation [101]. The involvement of Trpv4 in altering BBB permeability was further evidenced by Jie et al. [103], with pharmacological effects of the potent and specific TRPV4 agonist GSK1016790A. Once administered by ICV injection in mice, GSK1016790A triggered BBB disruption with markedly increased expression and activity of metalloproteinase-9 (MMP-9) and decreased protein expression of ZO-1 and occludin at the BBB. This effect was reversed by co-treatment with the TRPV4 antagonist HC-067047 [103]. Enhanced expression and activity of MMP-9 might more easily digest the brain capillary endothelial basal lamina and decrease ZO-1 and occludin protein expression, leading to BBB disruption [103].

The TRPV4 agonist 4αPDD caused a 3.4-fold increase in microvessel density in the ischemic region in rats bearing middle cerebral artery occlusion (MCAO) surgery, suggesting the possible involvement of TRPV4 in capillary formation and possibly BBB maturation [129]. Xie et al. have further showed that the significant increased BBB permeability during cerebral ischemia–reperfusion in a rat stroke model was ameliorated by administration of HC067047 [104]. The Evans blue content in the brain, as a measure of BBB disruption, was significantly decreased in the ischemia stroke rats receiving HC-067047 compared with the sham group [104]. Blockage of Trpv4 by HC-067047 reversed BBB disruption and inhibited brain edema in mice bearing MCAO surgery [103]. The brain water content and Evans blue brain extravasation at 48 h post-MCAO were significantly reduced by HC-067047, as well as the expression and activity of MMP-9 [103]. Spontaneous ICH has been regarded as a fatal stroke subtype [130], with 50% of ICH patients dying in the first 48 h [131]. BBB disruption is one of the main reasons for ICH [132]. Thus, a pharmacological blockade of TRPV4 would be a possible strategy for managing ICH treatments via attenuating BBB disruption. Trpv4 inhibition by its specific antagonist HC-067047 or Trpv4 knock-down dramatically ameliorated BBB disruption after ICH, as well as neurological functions, brain edema, and neuronal apoptosis [101]. Evans blue brain extravasation was decreased and the expression of adherens and TJ proteins was preserved in ICH rats under Trpv4 blockade, which was related to the decreased formation of stress fibers [101].

The mechanisms underlying TRPV4-mediated BBB permeability modulation are probably complex. Firstly, Ca^2+^ influx, which is elicited by activation of endothelial TRPV4 channels, can further activate intermediate and small conductance Ca^2+^-sensitive K^+^ (KCa) channels [114], while KCa channels have been well known to be able to induce BBB opening [115] (Figure 1). Secondly, increased intracellular Ca^2+^ via TRPV4 activation can also significantly induce the endothelial nitric oxide synthase (eNOS) phosphorylation and expression by over two-fold in the ischemic tissue [105] (Figure 1). Activated eNOS produces more nitric oxide (NO), further activating soluble guanylyl cyclase to produce more cyclic guanosine monophosphate (cGMP); high concentrations of cGMP lead to an increase in the number of pinocytic vesicles containing caveolin-1 and caveolin-2 and BBB permeability [116]. In fact, the mRNA and protein levels of caveolin-1 and caveolin-2 expression at mRNA and protein levels were significantly upregulated in cerebral microvessels of the ischemic area, which can be downregulated by HC-067047 [104]. Thirdly, another possible mechanism involved in TRPV4 activation mediated BBB disruption is the formation of stress fibers through the PKCα/RhoA/MLC2 pathway activation (Figure 1). PKCα activation has been involved in TRPV4-regulated endothelial cell permeability alteration [117]. After activation, PKCα can directly interact with RhoA, leading to the rearrangement of the cytoskeleton [118,119]. RhoA further binds to Rho-kinase, which can strongly induce the phosphorylation of myosin light chain 2 (MLC2) and the formation of actin stress fibers [120], thus leading to BBB disruption [121]. Therefore, TRPV4 antagonism is able to inhibit actin stress fibers formation in ischemia, thus preserving/rescuing the integrity of the BBB [101].

Trpv4 protein levels determined by Western blots were four-fold higher in the ipsilateral rat brain hemisphere bearing ICH surgery and a two-fold higher immunofluorescence Trpv4 signal was detected on the vascular structures [101]. The protein levels of Trpv4 in the ipsilateral hippocampus increased along with ongoing ischemia–reperfusion in mice bearing MCAO [133]. Higher expression and activity of TRPV4 most likely happens during cerebral ischemia–reperfusion. Therefore, the strategy of inhibiting TRPV4 has been demonstrated to possess neuroprotective effects in treating cerebral ischemic injuries in both in vitro and in vivo experiments [86,133]. Currently, many diseases are associated with TRPV4 mutations in humans, which are mainly related to various abnormal neurologic and/or musculoskeletal symptoms, including Charcot–Marie–Tooth disease type 2C [134], scapuloperoneal spinal muscular atrophy [135], congenital distaspinal muscular atrophies [136], autosomal dominant brachyolmia [137], and spondylometaphyseal dysplasia Kozlowski type [138]. Trpv4 KO mice results demonstrated a possible role of Trpv4 inhibition in preserving microcirculation and BBB/GVU function in mice bearing MCAO surgery [139]. In contrast to WT mice post MCAO surgery, Trpv4 KO mice showed reduced edema and Evans blue leakage, as well as milder neurological symptoms [139]. The loss of ZO-1 and occludin proteins in the ischemic hemisphere was also attenuated in Trpv4 KO mice [139]. Moreover, transmission electron microscopy study revealed that parenchymal microvessels in the ischemic lesion were narrowed and compressed by the swollen endfeet of astrocytes in wild-type mice post MCAO, but these effects were ameliorated in Trpv4 KO mice [139].

## 6. Discussion

BBB/GVU is vital to maintain brain homeostasis. BBB/GVU dysfunction, as a cause or consequence, is involved in various CNS diseases. A growing number of studies have targeted on BBB/GVU modulation and remodeling to treat CNS diseases. In this review, we summarize the expression of thermosensitive TRPV channels in the GVU and their possible role in modulating BBB/GVU properties. Among TRPV isoforms, TRPV2 and TRPV4 are the two main isoforms at the BBB, in contrast to the less expressed TRPV1 and the absence of TRPV3. TRPV2 is evidenced to play a main role in regulating the human BBB permeability, which enables the potential participation of TRPV channels in treating BBB dysfunction related to brain diseases, including, but not limited to, ischemic stroke.

More studies are needed to better establish the pathophysiological functions of TRPV in astrocytes and pericytes. Astrocytes are the most abundant glial cells and their endfeet surround the cerebral vascular system and release various molecules, further modulating the vascular tone and BBB/GVU functionality. Early evidence has suggested the possible involvement of astrocytes in modulating BBB/GVU via TRPV receptors. For example, Trpv1 and 4 was found specifically highly expressed in astrocyte endfeet facing brain microvessels [71,80]. Moreover, TRPV4 mediated Ca^2+^ mobilization in astrocyte endfeet was identified to contribute to neurovascular coupling, further mediating vascular tone and controlling the local perfusion in the brain [81,82]. Currently, the role of TRPV1 and TRPV2 in astrocyte–vascular coupling still remains to be explored. Mural cells (i.e., pericyte and smooth muscular cells) were evidenced to be important regulators in BBB/GVU functionality and hemodynamics. However, in contrast to astrocytes, even less information was known for the expression and role of TRPV in these mural cells.

TRPV1 or TRPV4 inhibition has been applied in treating ischemic stroke via preserving the BBB permeability in various in vitro and/or in vivo experiments, while TRPV2, the most expressed isoform in human BBB, is much less studied. This neglected role of Trpv2 using in vivo rodent models might be largely due to the less expression of Trpv2 in rodent BBB, which has been revealed by a recent study [66]. TRPV2 was the only identified isoform in human BBB using a non-targeted proteomic technique and was evidenced to be highly expressed in human BBB at gene and protein level [45]. In vitro studies using human BBB models revealed its role in controlling the proliferation, migration, tubulogenesis, and integrity of hBMECs [45]. The role of TRPV2 in physiological and pathological BBB/GVU deserves further be elucidation using in vivo non-rodent models, such as monkey and zebra fish, or genetically edited rodent models. These interspecies molecular differences between rodents and humans concerning the expression of TRPVs should be the subject of particular attention in the conclusions of experiments carried out in rodents concerning the expected effects in humans.

The pharmacological evaluations of TRPVs also require the use of old or new compounds whose pharmacokinetic properties make it possible to reach the brain parenchyma. Indeed, the BBB is often an obstacle because it limits the cerebral transport of many drugs and often represents an additional difficulty in such development and pharmacological evaluation of CNS targets. Interestingly, CBD is a drug recently approved for the treatment of epilepsy and is able to reach the brain parenchyma to exert its pharmacological effects. CBD, at low doses near pharmacological serum concentration in humans (0.3 to 3.2 µM), was shown to protect the BBB in vitro via a TRPV2 mechanism [140]. Apart from TRPV2, CBD was also reported to activate TRPV1 and TRPV4. However, the possible effects of CBD in modulating BBB/GVU and neurovascular coupling via TRPV are still lacking. CBD is a main natural compound isolated from *Cannabis sativa*. In contrast to THC, CBD does not produce psychoactive effects as it is neither a substrate of cannabinoid receptors CB1 nor CB2, and it has proven to be well tolerated in humans [141]. CBD at higher concentration (30 µM) induced cell death in human T24 bladder cancer cells through activating TRPV2 [142], and CBD decreased glioma stem-like cell viability with an IC50 of nearly 20 µM in a TRPV2-dependent manner [79]. CBD could also reduce lipopolysaccharide (LPS)-mediated BBB disruption [123] and protect BBB integrity in multiple sclerosis mice models [143]. Some studies also demonstrated that CBD had numerous anti-apoptotic and neuro-protective effects in animal models of brain inflammation, epilepsy and multiple sclerosis [144]. It is still an open question whether these effects were related to CBD-induced TRPV activation. 

The effect of the acute and chronic pharmacological modulation of TRPVs should also deserve further in vivo studies to evaluate both the innocuity and benefit of such strategy. Various first-generation antagonists of TRPV1, such as AZD1386 [28] and mavatrep [30], were terminated in clinical trials due to safety concerns such as increased body temperature [145] and loss of perception to heat and pain [146]. Interestingly, CBD which has been approved by the US and European drug agencies in 2018 (trade name Epidiolex^®^) to treat pharmacoresistant epilepsia in patients of 2 years of age and older demonstrates the feasibility of TRPV modulation with acceptable side effects.

## 7. Conclusions

It is important to better elucidate the role of TRPVs in healthy and diseased BBB/GVU. Among TRPV isoforms, TRPV2 and TRPV4 are the two most expressed isoforms in the BBB, in contrast to the less expressed TRPV1 and the absence of TRPV3. A TRPV2 agonist could exert a protective effect on CNS-affecting diseases via preserving or rescuing BBB integrity. However, the interspecies molecular differences between rodents and humans concerning the expression of TRPVs, at least in the BBB/GVU, should be the subject of particular attention in the conclusions and translational significance.

## Figures and Tables

**Table 1:** Overview of clinical trials and approved drugs targeting transient receptor potential vanilloid (TRPV)1–4.

Drug/Agent	TRPV Modulation	Disease	Clinical Stage	References
Capsaicin 8% topical patch (Qutenza^®^)	TRPV1 agonist	Neuropathic pain associated with postherpetic neuralgia and diabetic peripheral neuropathy of the feet	Approved in 2009	[26]
Capsaicin (Intra-articular injection)	TRPV1 agonist	Osteoarthritic pain	Phase II	[31]
Resiniferatoxin	TRPV1 agonist	Osteoarthritic pain	Phase III	[32]
NEO6860	TRPV1 antagonist	Osteoarthritic pain	Phase II	[27]
AZD1386	TRPV1 antagonist	Gastroesophageal reflux disease	Phase II	[33]
AMG-517	TRPV1 antagonist	Osteoarthritic pain	Phase II	[28]
SB705498	TRPV1 antagonist	Non-allergic rhinitis	Phase II	[34]
Mavatrep	TRPV1 antagonist	Osteoarthritic pain	Phase I	[30]
MK-2295	TRPV1 antagonist	Postoperative dental pain	Phase II	[35]
DWP05195	TRPV1 antagonist	Postoperative dental pain	Phase II	[36]
GRC15300	TRPV3 antagonist	Neuropathic pain	Phase II	[29]
GSK2798745	TRPV4 antagonist	Pulmonary edema, congestive heart failure	Phase II	[37,38]

**Table 2:** Overview of TRPV1–4 brain expression and modulators.

Subtype	Chemical Agonist	Chemical Antagonist	Ethanol	Proton	Temperature	Osmotic Stress	Shear Stress	Brain Expression/Distribution	References
TRPV1	Capsaicin, anandamide, 2-Arachidonoylglycerol, 2-APB, Camphor	Capsazepine, SB366791, SB705498, Ruthenium Red	+	+	>43 °C	Hyperosmotic Stress	-	Highly restricted brain expression: mainly in the caudal hypothalamus, the dorsal motor nucleus of the vagus, mesencephalic trigeminal nucleus, and parabrachial nucleus	[50,51,59,60]
TRPV2	Cannabidiol, 2-APB	Tranilast, Probenecid, Ruthenium Red	-	-	>53 °C	Hypoosmotic Stress	+	Intensive expression can be seen mainly in several brain areas, including hypothalamus, the nucleus of the solitary tract, hypoglossal nucleus, and the rostral division of the ventrolateral medulla	[37,45,52]
TRPV3	Camphor, Carvacrol, Eugenol, 2-APB	GRC15300, Ruthenium Red	-	-	>24 °C	-	-	Expressed in several brain areas, including hippocampus, pyramidal neurons, dorsal medulla, central nucleus of the amygdala, and the lateral septum	[52,53,54,55]
TRPV4	GSK1016790A, 4αPDD, Carbachol	HC-067047, RN-1734, Ruthenium Red	-	+	>27 °C	Hypoosmotic Stress	+	Widely expressed in the brain, especially in hippocampus, hypothalamus, cerebellum, cortical astrocytes, lamina terminalis, optic chiasm	[50,56,57,61]

**Table 3:** TRPV1–4 Expression and Function at the blood–brain barrier (BBB).

Studies	Species	BBB Source	Existence	Gene/Protein Analysis	Functional Stimulation	Functional Inhibition
*TRPV1*						
Golech et al. [44]	Human	Cultured BMECs (Brain microvascular endothelial cells, passage 7–10) isolated from micro vessels/capillaries	+	RT-PCR (Reverse Transcription-polymerase Chain Reaction), IF (Immunofluorescence)	Capsaicin; AM404; endocannabinoids including 2-arachidonoyl-glycerol (2-AG), anandamide (ANA) and methanandamide (m-ANA) (37 °C, 30 s)	Capsazepine; Ca^2+^-free medium
Hu et al. [62]	Rat	Isolated single venular capillaries (transendothelial electrical resistance (TEER) value 2400 Ω cm^2^)	+	-	Capsaicin (37 °C, 60 s)	Capsazepine
Brown et al. [63]	Mouse	1. Isolated mouse brain micro vessels; 2. bEnd.3 cells	-	RT-PCR	-	-
Hatano et al. [44]	Human	Cultured BMECs	-	RT-PCR, q-PCR	-	-
Zhang et al. [64]	Mouse	Isolated BMECs	+	RNA Sequencing	-	-
Yang et al. [65]	Mouse	1. In situ identification in murine capillaries marked by CD31; 2. bEnd.3 murine BBB cell line	+	1. IF; 2. IF, WB (Western blot)	-	Capsazepine
Luo et al. [66]	Human and Rat	1. Human: primary culture of isolated BMECs; 2. Human BBB cell line hCMEC/D3; 3. Rat: isolated capillaries and primary culture of isolated BMECs	+	q-PCR	-	Capsazepine
*TRPV2*						
Brown et al. [63]	Mouse	1. Isolated mouse brain micro vessels; 2. bEnd.3 cells	+	RT-PCR	Hypoosmotic stress	Ruthenium Red; Ca^2+^-free medium containing EDTA
Hatano et al. [46]	Human	Cultured BMECs	+	RT-PCR, qPCR	-	-
Zhang et al. [64]	Mouse	Freshly isolated BMECs	+	RNA Sequencing	-	-
Luo et al. [45]	Human and Rat	1. Human: primary culture of isolated BMECs; 2. Human BBB cell line hCMEC/D3; 3. Rat: isolated capillaries and primary culture of isolated BMECs	+	q-PCR, IF, WB	Cannabidiol (37 °C, 10 min); Heat	Tranilast; Ruthenium Red; siRNA targeting TRPV2
*TRPV3*						
Brown et al. [63]	Mouse	1. Isolated mouse brain micro vessels; 2. bEnd.3 cells	-	RT-PCR	-	-
Hatano et al. [45]	Human	Cultured BMECs	-	RT-PCR	-	-
Zhang et al. [64]	Mouse	Freshly isolated BMECs	-	RNA Sequencing	-	-
Luo et al. [66]	Human and Rat	1. Human: primary culture of isolated BMECs; 2. Human BBB cell line hCMEC/D3; 3. Rat: isolated capillaries and primary culture of isolated BMECs	-	q-PCR	-	-
*TRPV4*						
Brown et al. [63]	Mouse	1. Isolated mouse brain micro vessels; 2. bEnd.3 cells	+	RT-PCR	Hypoosmotic stress; 4αPDD (37 °C)	Ruthenium Red; Ca^2+^-free medium containing EDTA
Sullivan et al. [67]	Human	Cultured BMECs (passage 3–4)	+	RT-PCR, IF	GSK1016790A; 4αPDD (37 °C, 0.03–0.57 s)	HC-067047
Hatano et al. [46]	Human	Cultured BMECs	+	RT-PCR, qPCR, IF	Hypoosmotic stress; 4αPDD (37 °C)	Ruthenium Red; Ca^2+^-free medium containing EDTA; siRNA targeting TRPV4
Zhang et al. [64]	Mouse	Freshly isolated BMECs	+	RNA Sequencing	-	-
Harraz et al. [61]	Mouse	Freshly isolated BMECs	+	-	GSK1016790A; 4αPDD; Carbachol (37 °C)	HC-067047; Ruthenium Red; TRPV4-KO mice
Luo et al. [66]	Human and Rat	1. Human: primary culture of isolated BMECs; 2. Human BBB cell line hCMEC/D3; 3. Rat: isolated capillaries and primary culture of isolated BMECs	+	q-PCR, IF, WB	GSK1016790A(37 °C, <2 min)	RN-1734; Ruthenium Red

**Table 4:** Drugs/Agents targeting TRPV channels in modulating BBB integrity.

Drug/Agent	Dose	Route	Species	Model	Effect on BBB Integrity	Effect on TRPV Channels	Studies
Capsaicin	100 μM	ex-vivo	rat	Naive	Increase the permeability	TRPV1 Agonist	Hu et al. [62]
Capsazepine	1 μmol/kg	IP	rat	Experimental cerebral ischemia–reperfusion by middle cerebral artery occlusion (MCAO)	Reduced the permeability increase in brain capillaries induced by MCAO surgery	TRPV1 Antagonist	Hu et al. [62]
Capsazepine	35 nmol/L	Intra-cerebroventricular (ICV) injection	rat	Cerebral edema	Reduced the cerebral edema	TRPV1 Antagonist	Cernak et al. [98]
Capsazepine	1 μmol/kg	IP	rat	Experimental cerebral ischemia–reperfusion (MCAO)	Protect the BBB by limiting post-ischemic permeability increase	TRPV1 Antagonist	Gauden et al. [99]
Capsazepine	0.5, 1, 5, 10 mΜ	In vitro assay, in culture media	mouse	In vitro bEnd.3 cell line under stretch injury simulating traumatic brain injury (TBI)	Reduced the loss of ZO-1 induced by stretch injury in bEnd.3 cells	TRPV1 Antagonist	Yang et al. [93]
Capsazepine	1 μmol/kg, twice daily	IP	rat	TBI	Decreased the vasogenic brain edema and BBB disruption (Evans Blue extravasation), and preserved tight-junctions (TJ) upon brain injury	TRPV1 Antagonist	Yang et al. [65]
Cannabidiol	1 μM	In vitro assay, in culture media	human	In vitro BBB transwell model using primary brain microvascular endothelial cells (PBMECs)	Increased the TEER value of hPBMEC monolayers cultured in transwell	TRPV2 Agonist	Luo et al. [45]
Cannabidiol	10 μM	In vitro assay, in culture media	human	In vitro BBB model consisting by human PBMECs under oxygen-glucose deprivation (OGD) simulating ischemic stroke	Prevented the increase in BBB permeability induced by OGD	TRPV2 Agonist	Hind et al. [100]
GSK1016790A	50 pmol per rat	ICV injection	rat	Naive	Induced adherens and TJ protein degradation	TRPV4 Agonist	Zhao et al. [101]
4α-PDD	100 nM	In vitro assay, in culture media	mouse	In vitro BBB transwell model using bEnd3 cell monolayers	Increased the permeability of bEnd3 cell monolayers cultured in transwell	TRPV4 Agonist	Brown et al. [63]
4α-PDD	0.1 mg/kg	IV injection	rat	Experimental cerebral ischemia–reperfusion (MCAO)	Caused a 3.4-fold increase in microvessel density in the ischemic region in rats bearing MCAO surgery	TRPV4 Agonist	Chen et al. [102]
HC-067047	5, 50, 150 pmol per rat	ICV injection	rat	Autologous arterial blood was injected into the basal ganglia area to mimic ICH	Preserved the expression of adherens and tight junction (TJ) protein, as well as BBB integrity (Evans Blue extravasation) during ICH	TRPV4 Antagonist	Zhao et al. [101]
HC-067047	10 μmol per mouse	ICV injection	mouse	Experimental cerebral ischemia–reperfusion (MCAO)	Reversed BBB disruption and inhibited brain edema and infarction after MCAO surgery	TRPV4 Antagonist	Jie et al. [103]
HC-067047	50 pmol per rat	ICV injection	rat	Experimental cerebral ischemia–reperfusion (MCAO)	Reduced the BBB permeability increase induced by MCAO surgery	TRPV4 Antagonist	Xie et al. [104]

## Data Availability

Not applicable.
